# Addressing Cancer Health Disparities in the Pacific Peoples of Hawai‘i, Guam, and the US Associated Pacific Islands Through Pacific-Focused Research Capacity Building

**DOI:** 10.1200/GO.19.00213

**Published:** 2020-01-13

**Authors:** Rachael T. Leon Guerrero, Neal A. Palafox, Margaret P. Hattori-Uchima, Hali R. Robinett, Carl-Wilhelm Vogel

**Affiliations:** ^1^ University of Guam, Mangilao, GU; ^2^ University of Hawai‘i Cancer Center, Honolulu, HI

## Abstract

Sociocultural, geographic, and biologic factors contribute to cancer health disparities (CHDs) in Indigenous Pacific peoples (IPPs) in Guam, Hawai‘i, and the US Associated Pacific Islands (USAPI). IPPs experience a greater burden of CHDs that are associated with late-stage diagnosis and poor survival outcomes compared with majority populations in the United States. A 16-year partnership between the University of Guam (UOG) and University of Hawai‘i Cancer Center (UHCC) aims to advance health equity in Guam, Hawai‘i, and the USAPI through cancer research, training, and outreach. Investigators at collaborating institutions study issues of regional and cultural relevance in IPPs, including breast, cervical, liver, and oral cancers and use of tobacco and betel nuts (Areca nuts). Junior faculty with IPP ancestry or those who are focused on CHDs in IPPs receive mentorship and career development opportunities, academic fellowships are provided for graduate students, and Pacific Island communities are engaged through a participatory development process. The partnership has generated more than 90 peer-reviewed publications, more than 100 abstracts, and 11 grant awards. Thirty graduate scholars from under-represented minorities have been trained, including two who are now UOG faculty and are conducting independent research, contributing to the partnership, and mentoring scientists of tomorrow. Participatory community engagement has contributed to the passage of significant cancer prevention and control legislation in Hawai‘i, Guam, and Saipan. Research capacity at UOG has increased significantly, and research at UHCC has expanded to address issues unique to IPPs. Graduate students from under-represented minorities are pursuing careers in cancer research. A regional research infrastructure has been established to support team science, and research findings are informing public health policy and planning.

## INTRODUCTION

Cancer health disparities (CHDs) is a term used to describe the unequal burden of cancer incidence, morbidity, and mortality among different populations. Higher and unequal burdens of cancer are typically observed in ethnic minorities, immigrant communities, inner city populations, and rural, insular, or geographically isolated populations.^1,2^ There are many factors contributing to CHDs, including genetic makeup, cultural norms and beliefs, behavior, environmental factors, and social determinants of health such as socioeconomic status, poverty, and education. Reducing CHDs is an important and integral component of the nation’s effort to improve the health of all Americans, including those with Pacific Islander ancestry.

**CONTEXT**

**Key Objective**
A partnership between the University of Guam and the University of Hawai‘i Cancer Center continues to advance health equity in Guam, Hawai‘i, and the USAPI through cancer research, training, and outreach.
**Knowledge Generated**
Investigators at collaborating institutions research issues of regional and cultural relevance in Indigenous Pacific people.
**Relevance**
Underrepresented graduate students are pursuing careers in cancer research, and regional research infrastructure has been established and strengthened.

The Pacific Ocean connects approximately 10,000 islands and extends between Asia and America. The islands make up Oceania (a term used by the WHO), and they are divided into three subregions known as Polynesia, Melanesia, and Micronesia.^3^ The Indigenous Pacific peoples (IPPs), classified by historical migration and linguistic similarities are referred to as Polynesians, Melanesians, and Micronesians. The subregional populations each have several culturally and linguistically distinct island groups. The Native Hawaiian population (the largest US based Indigenous Pacific population from Hawai‘i) and other IPPs whose countries are politically linked to the United States are the focus of this work. Within the last five decades, the health of the IPP populations has dramatically shifted, and now those populations have one of the highest incidences in the world of preventable chronic diseases such as obesity, diabetes, and cancer.^4^ The disparate health outcomes of IPPs can be attributed to a multitude of complex socioeconomic factors. The IPP population generally has higher poverty rates, lower income, larger families, and lower educational status.^5^ According to the 2015 US Census Bureau report, 17.3% of IPPs were living in poverty compared with 10.4% of non-Hispanic whites. IPPs also have lower educational attainment with only 28% of the population earning a bachelor’s degree or higher compared with whites of whom 47% obtain a bachelor’s degree or higher.^6^

IPPs from the Hawai‘i-Pacific region continue to experience significant CHDs with regard to cancer risk despite tremendous advancements in cancer research and scientific discovery. The existence of these disparities among specific IPP subgroups, such as Chamorros (Guam’s Indigenous population), and for numerous types of cancers are a result of lifestyle, genetic and environmental factors, limited cancer awareness and education, poor access to cancer screening, late-stage diagnosis, limited cancer treatment options, and thus poor treatment outcomes. For example, geographic, educational, and resource barriers contribute to the highest rates of cervical cancer in Micronesia where cervical cancer incidence in Micronesian women is eightfold higher than the national average (79.7 per 100,000 *v* 9.9 per 100,000). Cervical cancer incidence in Guam was nearly double the rate in Hawai‘i for the period 2009 to 2013.^7^ Compared with men in Hawai‘i, men in Guam, particularly Chamorros, are disproportionately affected by poor outcomes for a number of major cancers, including cancers of the lung and bronchus, nasopharynx, liver, and intrahepatic bile duct. Rates of liver and intrahepatic bile duct cancer among men in Guam were nearly double those of men in Hawai‘i and nasopharyngeal cancers were more than four times higher than those of men in Hawai‘i.^7^

In addition to being a highly under-represented and underserved minority with a significant burden of health disparities, IPPs also struggle with educational attainment. According to a report by the National Commission on Asian American and Pacific Islander Research in Education (2013), educational attainment of the younger generation (age 25 to 34 years) of Native Hawaiians and Chamorros is comparatively less than that of the older generation (age 55 to 64 years) of these populations. IPPs, specifically Micronesians, are also under-represented in the biomedical research community. According to a report by the American Council on Education, only 20.4% of IPPs nationwide enroll in college to pursue a degree, and of those, only 20% pursue a bachelor’s degree in a science, technology, engineering, or math (STEM) field.^8^ A survey of doctoral degrees awarded in biological sciences in a 1-year period in the United States showed that not a single Native Hawaiian or other Pacific Islander received a PhD during that time frame.^9^ This alarming trend of downward educational mobility is a clear demonstration of disparity for IPPs and speaks to the need for increased funding and opportunities for these populations.

**FIG 1:**
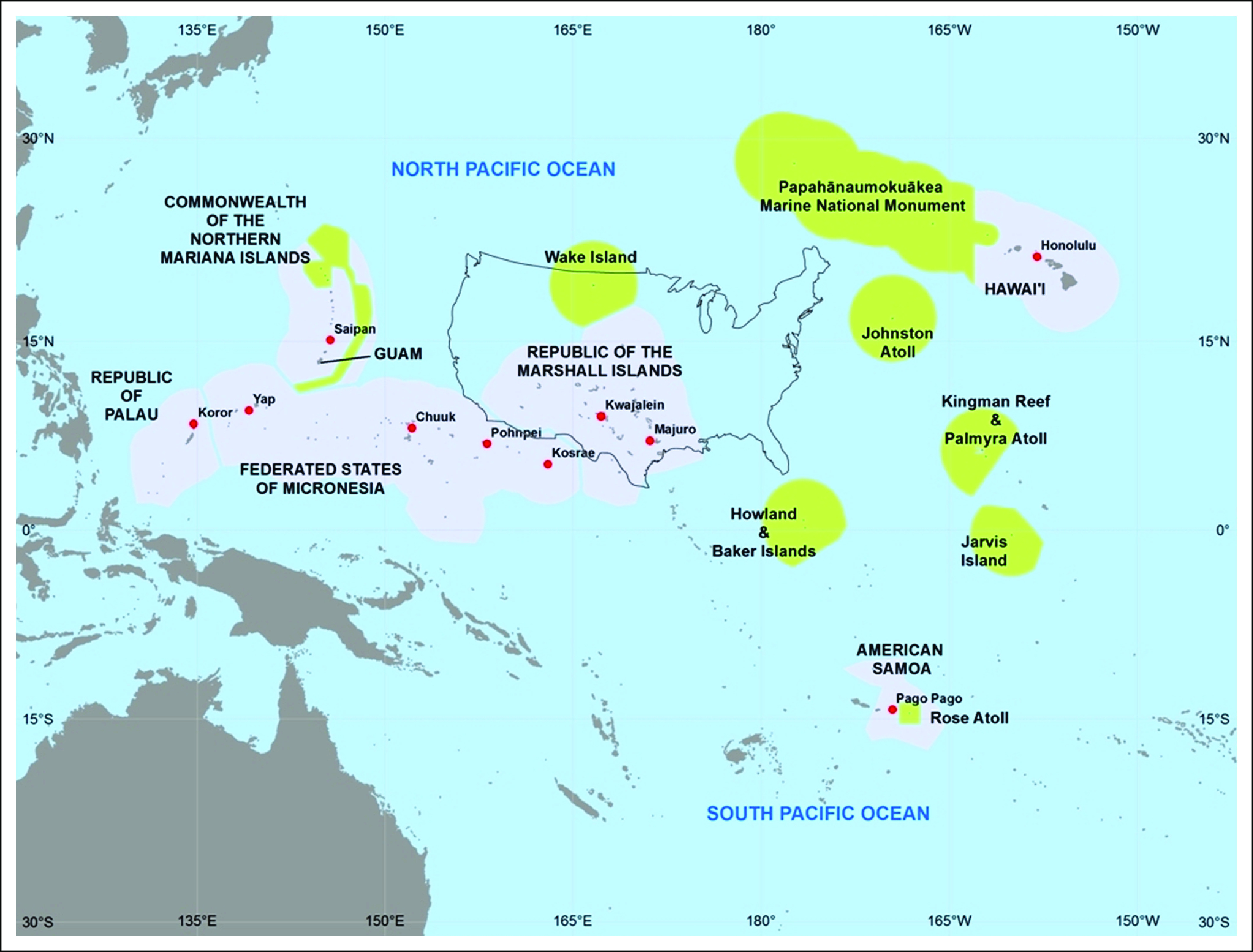
Map of the Pacific.

In 2019, the University of Guam (UOG) and University of Hawai‘i Cancer Center (UHCC) marked 16 years of continuous funding awarded by the National Cancer Institute (NCI) to support collaborative research, training, and outreach to address CHDs in Americans of Indigenous Pacific ancestry. The UOG/UHCC Partnership is one of only 14 Partnerships to Advance Cancer Health Equity (PACHE), a program of the NCI that, since 2001, has supported the coming together of NCI-designated cancer centers and minority-serving institutions for the express purpose of studying CHDs and their impact on racial/ethnic minorities and medically underserved and socioeconomically disadvantaged populations. The UOG/UHCC Partnership is the only PACHE addressing CHDs in IPPs, with a special focus on Micronesians. The Partnership is characterized by a unique geography. The region encompasses Hawai‘i, Guam, the Commonwealth of the Northern Mariana Islands, and the other US Associated Pacific Islands (USAPI) jurisdictions—a region larger than the continental United States (Fig 1). The two partner organizations are separated by 3,800 miles, four time zones, and the international dateline. The island jurisdictions are distant, fragmented, and isolated. Access to health care services and information is limited, and the health care infrastructure is inferior compared with those in Hawai‘i and the US mainland. Despite these challenges, the Partnership aims to advance health equity in Guam, Hawai‘i, and the USAPI through development of cancer research capability and faculty competitiveness at UOG and UHCC. The Partnership is particularly focused on CHDs of regional relevance, raising awareness of cancer, and cancer prevention in the multiethnic communities served by UOG and UHCC, and increasing the number of cancer and biomedical science researchers who study IPP ancestry by offering graduate training programs for IPP students in cancer-related fields. The purpose of this article is to highlight the efforts of the U54 Partnership between UOG and UHCC and showcase the impacts that such a partnership can have in a community of underserved and under-represented people.

## RESEARCH

Through the UOG/UHCC Partnership, NCI PACHE has provided long-term infrastructure support for cancer research in the Hawai‘i-Pacific region, graduate training in CHDs, mentoring and career development for early-stage investigators (ESIs) at the partnering institutions, outreach education for communities in Guam, Hawai‘i, and the USAPI, and funding for new and innovative cancer research projects of particular relevance to Hawai‘i, Guam, and the USAPI.

Investigators at collaborating institutions study issues of regional and cultural relevance in IPPs, such as breast, cervical, liver, and oral cancers and tobacco and betel nut (Areca nut) use. The Partnership has a clear criteria and review process for awarding research projects. At least once a year, Partnership leaders issue a call for proposals and encourage new investigators to first submit a short 1-page concept of their proposed project. The Internal Advisory Committee of the Partnership reviews these concepts and determines which of the researchers who submitted a concept proposal will be invited to submit a full proposal. Researchers are invited on the basis of the scientific merit and alignment of their concepts with Partnership goals. The investigators are then asked to submit a complete proposal. Once a complete proposal is formally submitted, the Internal Advisory Committee members will review it and make recommendations on funding. In addition to the standard criteria typically used for National Institutes of Health research grants (significance, methodologic approach, environment, investigators, and innovation), reviewers also evaluate proposals by using the following additional criteria: the relationship of the project to the overall objectives of the Partnership and other funded projects in the Partnership, the potential to develop into a project with reasonable future chances of being funded from peer-reviewed sources, and adequate description of research enrichment activities in case ESIs are involved.

In the current U54 funding cycle (2015-2020), the UOG/UHCC Partnership has focused on two regionally relevant areas of research; betel nut use and cervical cancer. Betel nut is the fourth most commonly used psychoactive substance in the world and is chewed for cultural and religious reasons and as a stimulant by more than 600 million people concentrated in South and Southeast Asia and in the Western Pacific, including the U.S. Territory of Guam, the U.S. Commonwealth of the Northern Mariana Islands, the Republic of Palau, and the Federated States of Micronesia, notably the Yap and Chuuk states. The practice is spreading eastward in the Pacific as Micronesians migrate to neighboring jurisdictions, including Guam and Hawai‘i where betel nut is locally harvested from the Areca palm tree and sold by neighborhood vendors catering to Asian and Pacific Island communities. Betel nut is classified by the International Agency for Research on Cancer as a Group 1 carcinogen associated with oral, oropharyngeal, and esophageal cancer, as well as oral legions, gum disease, and oral submucosal fibrosis. When chewed in combination with tobacco, as is commonly the practice, betel nut is also linked to cancers of the pharynx. Other additives that vary by region include the Piper betel leaf, calcium hydroxide, alcohol, and spices.

## RESEARCH EDUCATION

Cancer health equity in IPPs is difficult to realize without research designed and conducted by, for, and with IPPs. The UOG/UHCC Partnership provides support through its Research Education Core for ESIs and graduate students from under-represented minorities in the form of training in CHD research and mentoring and career development at the partnering institutions.^10^ At UOG, up to four master’s degree students per year are awarded 2 years of support to pursue degrees in existing graduate programs. All master’s degree students supported by the Partnership are required to complete a research thesis that is focused on CHDs. In addition, U54-supported master’s degree scholars are exposed to and engaged in all currently funded U54 research, a requirement of their award. At UHCC, the Partnership supports up to two doctoral students each academic year, one supported by U54 funds and another supported by UHCC institutional funds dedicated to the U54. Together with their mentors, U54 master’s degree and doctoral degree scholars are expected to create a career development plan and to present their research thesis to the U54 team before completing their program. UOG and UHCC U54 graduate students, those awarded graduate fellowships, and others hired by investigators of U54-sponsored research are encouraged to participate in additional courses and activities to strengthen their ability to conduct research related to cancer and health disparities in preparation for health and research careers in the Pacific region. Seven online training modules have been developed by UOG and UHCC faculty to support the training aims of the Partnership.

The partnership aims to support and develop junior faculty from under-represented minority ESIs at UOG and UHCC, especially those of IPP ancestry, who want to conduct research in CHDs. ESIs are mentored by experienced U54 faculty to strengthen their research skills, such as skills in research design, grant writing, manuscript development, navigating the institutional review board process, and working with community advisory boards and other areas related to conducting, analyzing, and disseminating CHD research. ESIs are informed about and encouraged to participate in research training and career development opportunities made available through the Partnership, the partnering institutions, the National Institutes of Health, and other sponsors, including the NCI-funded Geographical Management of Cancer Health Disparities Program (GMaP, Region 5), which sponsors an annual career development workshop.^11^ The Partnership also provides learning and career development opportunities through travel support for students and ESIs so they can make presentations at national conferences and meetings. The Partnership also provides summer travel and salary support for faculty, doctoral students, and ESIs so they can conduct research, prepare manuscripts, develop research proposals, and collaborate with faculty members at the partnering institutions on other research activities.

The support provided for ESIs and graduate students to engage in CHD research addresses the Partnership’s overarching goal of developing scientists from under-represented minorities in basic, clinical, translational, behavioral, and population research, while increasing the number of IPPs who have successful careers as health professionals and scientists who are committed to addressing cancer prevention and control priorities of island communities in the Pacific region.

## COMMUNITY OUTREACH

The Partnership’s Community Outreach Core has been responsive to the recommendations of the Community Advisory Groups at both UOG and UHCC and has engaged in community prevention and education activities related to relevant CHD issues. In Guam, outreach efforts have focused heavily on increasing uptake of human papilloma virus vaccination through provider education and community awareness initiatives. After the recent purchase of an inflatable colon for teaching purposes, UOG’s outreach team is leading efforts to promote colorectal screening in Guam. In Hawai‘i, UHCC’s outreach team is working with members of Oahu’s Kosraean community and health care providers at Kapiolani Medical Center for Women and Children in Honolulu to identify and implement strategies to facilitate mutually trusting relationships and the delivery of culturally competent care.

## OUTCOMES

Outcomes during the U54 funding period (since September 2009) include significant scientific discoveries published in more than 90 peer-reviewed publications, a list of which is provided on our Web site (http://www.guamcrc.org). Other outcomes include 11 grants awarded to U54 investigators, eight faculty exchange visits, and 22 master’s degree students and eight doctoral students supported for one or more semesters. They include four PhD graduates from Guam and other parts of Micronesia at UHCC, two of whom are now serving as faculty at UOG and contributing as U54 investigators and mentors of future scientists from Guam and other parts of Micronesia. In addition, a master’s degree level track in CHDs was developed at UOG in its Micronesian Studies Program, which has since been institutionalized at UOG.

To date, 12 betel nut studies have been supported by the UOG/UHCC Partnership, including three molecular studies, three population measures studies, three mechanistic studies, and two prevention studies, including an intervention trial. In the current funding cycle (2015-2020), the Partnership is supporting an adult betel nut cessation trial in Guam, which is the first known randomized intervention and is modeled after group tobacco cessation interventions. There is also a pilot study exploring betel nut biomarkers in urine and saliva to help validate betel nut use in humans, an oral microbiome study evaluating the influence of chewing betel nuts on oral bacterial composition plus bacterial composition of the betel nut and Piper betel leaf, and a study to identify the molecular components of betel nuts that promote chronic inflammation, which has an important role in carcinogenesis. The partnership’s investment in betel nut research is of global and regional importance because study findings will inform future public health interventions and clinical recommendations for betel nut users in the Pacific region and worldwide.^12^

Through community outreach program activities and collaborations with local organizations, the Partnership directly and indirectly had a beneficial impact on cancer awareness and public health policy on Guam, with long-lasting beneficial consequences for the Territory of Guam. In June 2006, the Guam Legislature passed Public Law 20-80, locally known as the Natasha Perez Protection Act, which made all enclosed public places on Guam smoke-free, including restaurants. In November 2009, the Guam Legislature passed Public Law 30-63, which prohibited smoking within 20 feet of an entrance to a public place where smoking is prohibited. In 2010, Guam Public Law 30-80 was enacted, which increased the tax on cigarettes by $2.00 per pack (the single largest tax increase for cigarettes ever enacted in the country) and placed Guam among the US jurisdictions with the highest tax on cigarettes. Significantly, all proceeds from tobacco taxes are deposited into the Healthy Futures Fund, not to be comingled with the Guam general fund. Fifteen percent of the taxes collected (approximately $1M annually) is transferred into the Guam Cancer Trust Fund for programs that support cancer screening, treatment, and support services, and one percent of the taxes collected is used to maintain the Guam Cancer Registry (GCR). In September 2016, through the work of U54 investigators studying betel nut use, legislation prohibiting the sale of betel nuts to minors was adopted in the neighboring Northern Mariana Islands.

The GCR, once an unfunded legislative mandate, was developed with help from the Hawai‘i Tumor Registry and UOG/UHCC Partnership. Before that, collecting cancer data on Guam was limited to analyses of death certificates by the Guam Department of Public Health and Social Services. Through Partnership funding, GCR was finally able to hire full-time personnel in 2003. They helped the GCR collect more accurate and complete information regarding Guam cancer cases by actively observing health care providers and helping them review patient records, obtain additional information, and periodically contact cancer survivors. In recognition of its progress, the GCR was awarded full-member status in the North American Association of Central Cancer Registries in 2006.^13^ In 2009, the GCR published its first comprehensive report, “Guam Cancer Facts & Figures 2003-2007.” In 2015, the GCR published its second report, “Guam Cancer Facts & Figures 2008-2012.” Partnership funding led to the development of a fully operational cancer registry, which is an important resource for research and public health in Guam. Long-term sustainability of the GCR was then achieved in 2010 when the Guam Legislature passed Public Law 30-80, which increased the tobacco tax and provided for a dedicated funding stream to support the operation of the GCR.^14^

In conclusion, infrastructure and resources for cancer research and surveillance were nonexistent in Guam before the UOG/UHCC Partnership was established in 2003. The Partnership has significantly increased research capacity at UOG and cultivated interest and engagement in cancer research among investigators from under-represented minorities and minority IPP students alike. Scientists from a variety of disciplines and departments at both institutions are collaborating to address CHDs in Guam, Hawai‘i, and the neighboring USAPI. IPP populations, particularly Micronesians in Guam and Hawai‘i, are more aware of the risks associated with cancer, and providers are becoming more sensitive to the needs of these patients. Although the partnership has made progress in its 16-year history, there is much more to be done to overcome the unequal burden of cancer in IPPs.
